# Crossing barriers: a multidisciplinary approach to children and adults with young-onset movement disorders

**DOI:** 10.1186/s40734-018-0070-x

**Published:** 2018-04-06

**Authors:** Martje E. van Egmond, Hendriekje Eggink, Anouk Kuiper, Deborah A. Sival, Corien C. Verschuuren-Bemelmans, Marina A. J. Tijssen, Tom J. de Koning

**Affiliations:** 1 Department of Neurology, University of Groningen, University Medical Center Groningen, Groningen, the Netherlands; 2 Department of Neurology, Ommelander Ziekenhuis Groningen, Delfzijl and Winschoten, PO Box 30.001, 9700 RB Groningen, the Netherlands; 3 Department of Pediatrics, University of Groningen, University Medical Center Groningen, Groningen, the Netherlands; 4 Department of Genetics, University of Groningen, University Medical Center Groningen, Groningen, the Netherlands

**Keywords:** Multidisciplinary, Young-onset movement disorders, Diagnosis, Dystonia, Myoclonus, Clinical phenotyping

## Abstract

**Background:**

Diagnosis of less common young-onset movement disorders is often challenging, requiring a broad spectrum of skills of clinicians regarding phenotyping, normal and abnormal development and the wide range of possible acquired and genetic etiologies. This complexity often leads to considerable diagnostic delays, paralleled by uncertainty for patients and their families. Therefore, we hypothesized that these patients might benefit from a multidisciplinary approach. We report on the first 100 young-onset movement disorders patients who visited our multidisciplinary outpatient clinic.

**Methods:**

Clinical data were obtained from the medical records of patients with disease-onset before age 18 years. We investigated whether the multidisciplinary team, consisting of a movement disorder specialist, pediatric neurologist, pediatrician for inborn errors of metabolism and clinical geneticist, revised the movement disorder classification, etiological diagnosis, and/or treatment.

**Results:**

The 100 referred patients (56 males) had a mean age of 12.5 ± 6.3 years and mean disease duration of 9.2 ± 6.3 years. Movement disorder classification was revised in 58/100 patients. Particularly dystonia and myoclonus were recognized frequently and supported by neurophysiological testing in 24/29 patients. Etiological diagnoses were made in 24/71 (34%) formerly undiagnosed patients, predominantly in the genetic domain. Treatment strategy was adjusted in 60 patients, of whom 43 (72%) reported a subjective positive effect.

**Conclusions:**

This exploratory study demonstrates that a dedicated tertiary multidisciplinary approach to complex young-onset movement disorders may facilitate phenotyping and improve recognition of rare disorders, with a high diagnostic yield and minimal diagnostic delay. Future studies are needed to investigate the cost-benefit ratio of a multidisciplinary approach in comparison to regular subspecialty care.

## Background

Young-onset movement disorders (YMDs) is a relatively new field in neurology, comprising clinical neurological syndromes presenting with involuntary movements manifesting before the age of 18. As with movement disorders (MDs) in adults, YMDs are subdivided into hyperkinetic movements (dystonia, myoclonus, chorea, ballism, tremor and tics), hypokinetic (parkinsonism) movements, and ataxia [1–5]. Recognition of common YMDs, such as tics and stereotypies, is usually straightforward for most clinicians. However, diagnosis of less common and more complex YMDs, such as disorders presenting primarily with myoclonus or dystonia, is often difficult, both for pediatric and adult neurologists [1, 6, 7].

The recognition and classification of YMDs present some unique challenges. Firstly, YMDs are often embedded in a complex clinical phenotype. For example, the occurrence of mixed MDs (more than one MD present) or co-existence of a variety of symptoms such as psychomotor retardation or behavioral abnormalities are commonly seen [5, 8, 9]. Secondly, in young children the developing nervous system may produce a variety of motor patterns that would be labeled as pathologic in older children and adults, but are simply a manifestation of brain immaturity in younger patients [1]. For instance, chorea is a normal feature in healthy infants and toddlers, and (subtle) signs of overflow dystonia and ataxia are found in healthy children up till the age of 12 years or even older [10, 11]. Finally, YMDs can be caused by a broad spectrum of both acquired and genetic disorders, including infections, auto-antibody and auto-immune disorders, as well as rare metabolic disorders and other inherited defects [7, 12–14].

The complexity of the diagnosis and management of YMDs is becoming increasing clear, which has resulted in a growing number of specialized pediatric neurologists. Despite this development, the diagnostic process in complex YMDs often remains challenging, a burden for patients and their families, and costly for our health care system as patients often remain undiagnosed for many years [1, 6, 7, 14, 15]. This has been reflected in a recent study in a tertiary referral center that showed a mean delay of diagnosis of 11.1 ± 12,5 years in a cohort of 260 patients with non-tic YMDs [7].

In other heterogeneous or rare diseases in children such as epilepsy or neuromuscular disorders, a beneficial effect of a multidisciplinary approach has been reported. [16–20] We hypothesized that such an approach might be a possibility to tackle the complexity of children and young adults with MDs. A multidisciplinary team may enable to overcome the three difficulties experienced in this patient group: a complex clinical phenotype (movement disorder specialist), the variety of motor patterns produced by the developing brain (pediatric neurologist), and a broad spectrum of both acquired and genetic disorders (pediatrician for inborn errors of metabolism and a clinical geneticist).

In this exploratory study, we report on the first 100 patients with YMDs who visited our multidisciplinary outpatient clinic. Our aim was to share our experience of a new multidisciplinary approach in terms of changes in MD classification, diagnostic yield and targeted treatment strategies.

## Methods

### Design and setting of the study

In this retrospective, single center, observational study we evaluated the first 100 patients who visited our multidisciplinary outpatient clinic for YMDs. It was situated in a tertiary referral center, the University Medical Center Groningen, in the Netherlands. The study was performed according to the ethical standards and regulations of our institute.

### Patients

All patients had a confirmed or suspected MD with an onset before the age of 18 years and were referred for an expert opinion regarding MD classification, etiology or treatment of involuntary movements (Table 1).

**Table 1:** Baseline characteristics

Patient characteristics	
Sex (male/female)	56/44
Age (years)^a^	12.5 ± 6.3; 1–33
Age at symptom-onset (years)^a^	3.3 ± 4.6; 0–18
Duration of symptoms (years)^a^	9.2 ± 6.3; 1–32
Referral questions	
Movement disorder classification	50
Etiology	38
Treatment	42
MD classification	
Ataxia	9
Dystonia	32
Myoclonus	11
Other^b^	12
Unclassified	36
Etiological diagnosis	
*Inherited etiologies*	17
Monogenic	
* ARX* mutation	1
Ataxia telangiectasia	1
Coffin Lowry syndrome	1
Glutaric aciduria type 1	2
* GLI2* mutation	1
* GOSR2* mutation	1
* GTPCH* deficiency (DYT5)	1
Proprionacidemia	1
* SCN1A* mutation	2
* THAP1* mutation (DYT6)	2
* TITF1* mutation	1
Structural chromosomal abnormality	
Microdeletion 19p13.2p13.13 (*NFIX* and *CACNA1A* gene)	1
Partial deletion chromosome 7q (*SCGE* gene)	1
Uniparental disomia chromosome 7 (*SCGE* gene)	1
*Acquired etiologies*	12
Infectious	2
Perinatal asphyxia	9
Functional	2

^a^Age in years ± standard deviation; range

^b^Chorea, tics, tremor, parkinsonism and if no MD was present

*Abbreviations: ARX*, Aristaless related homeobox; *GOSR2*, Golgi SNAP receptor complex member 2; *GTPCH*, Guanosine Triphosphate Cyclohydrolase; *SCN1A*, sodium channel voltage gated type I alpha subunit; *TITF1*, Thyroid transcription factor-1; *NFIX*, nuclear factor I/X; *CACNA1A*, calcium channel voltage-dependent, P/Q type, alpha 1A subunit; *SCGE*, epsilon-sarcoglycan

#### Multidisciplinary outpatient clinic

The clinic was initiated in 2012 with a team consisting of an adult neurologist specialized in MDs (MT), a pediatric neurologist specialized in developmental neurology and YMDs especially ataxia (DS), a pediatrician specialized in metabolic diseases (TK) and a clinical neuro-geneticist (CV). In addition, clinical fellows in movement disorders and residents attend the clinic to gain skills and knowledge from the different medical specialties involved as part of their clinical training.

The pediatrician for inborn errors of metabolism received the referrals as the coordinating medical specialist, which were subsequently discussed within the team. Prior to the consultation, referral letters and medical reports containing previous diagnostic and treatment strategies were read carefully by the clinical fellow, who sent a summary to all team members.

During the consultation, patients were seen by all team members at once. In a separate meeting, the team members reviewed the video images, discussed the movement disorder classification and the results of the additional investigations, and developed joint diagnostic and therapeutic recommendations. In all cases the team members reached consensus. The main diagnostic steps were laboratory investigations, (neuro-)imaging, clinical neurophysiology or genetic testing. The key therapeutic options comprised pharmacological treatment, botulinum toxin injections, paramedical interventions (e.g. physiotherapy, occupational therapy, speech therapy), ketogenic diet, and deep brain stimulation.

The primary purpose of the multidisciplinary team was not to take over the clinical care provided by the referring medical specialist, but preferably to see a patient once and provide an all-in-one expert opinion. The presence of the clinical geneticist enabled direct genetic counseling in case genetic testing was considered. Results of additional investigations and genetic diagnostics were shared with the patient or caregivers by one of the team members via a follow-up consult or, if preferred by the family, by a telephone consultation. The team aimed to leave further management and follow-up to the referring specialist, but in case of unresolved issues patients were welcome to return to the multidisciplinary outpatient clinic.

#### Data collection

We evaluated the first 100 patients who visited our multidisciplinary clinic for YMDs between June 2012 and May 2014. Medical records were reviewed for patient characteristics and previous phenotypical classifications. The severity of the YMDs present was assessed by the team members using the global clinical impression scale of severity (GCI-S). This commonly used 7-point scale enables a clinician to rate the extent movement disorders with no movement disorder (1), slight (2), mild (3), moderate (4), marked (5), severe (6), and among the most severest (7) [21]. We compared the classification of the most prominent MD and etiological diagnosis before and after assessment by the multidisciplinary team. In addition, we studied the treatment strategies and whether the patients or their caregivers reported any positive effects of therapies 3–6 months after initiation. Since many patients were not under our primary care, and/or living at a distance from our center, we performed follow-up using a semi-structured interview during a telephone consultation. Patients and/or caregivers were asked (1) whether they experienced benefit with regard to motor symptoms, (2) since when they experienced this, (3) extent of improvement (none/slight/moderate/good), and (4) if any adverse effects were present.

## Results

### Patient characteristics

A total of 56 male and 44 female patients visited the multidisciplinary clinic (Table 1). Patients had a mean age of 12.5 years (SD 6.3) and a mean duration of symptoms of 9.2 years (SD 6.3). Referring specialists were predominantly pediatric neurologists, pediatricians and rehabilitation doctors with questions concerning the MD classification, etiology or treatment options. We had 36 patients referred with an unclassified MD, documented as dyskinesias, trembling, involuntary movements, or restlessness. A confirmed etiological diagnosis (17 inherited, 12 acquired) already explained the phenotype of 29 patients upon referral.

### Movement disorder classification

Mean severity of the MDs present was 4.3 ± 1.7 on the global clinical impression scale (range 1–7), corresponding with a moderate to marked MD severity. The multidisciplinary team revised the initial classification in 58/100 patients (Table 2). These revisions reduced the number of patients with an unclassified MD from 36 down to 4. Compared to the referring clinicians, the team more frequently classified the patients’ involuntary movements as dystonia (from 32 to 41) and myoclonus (from 11 to 31). The number of ataxic and tremor patients dropped (from 9 to 1 and 6 to 1, respectively), whereas the number of patients with chorea increased (from 4 to 6). The multidisciplinary team observed no MDs in eleven patients (e.g. the movements were related to agitation or caused by behavioral abnormalities). Simultaneous non-invasive surface electroencephalography/electromyography (EEG/EMG) was performed in 29 predominantly myoclonic patients and this confirmed or supported the MD classification observed by the team in 24/29 patients. In the remaining five cases, EEG/EMG was not conclusive due to an absence of symptoms during registration (*n* = 3) or the patient being unable to comply with the registration protocol (*n* = 2).

**Table 2:** Overview of classification of most prominent MD before and after visiting the multidisciplinary outpatient clinic

		Observed MD classification by the multidisciplinary team					
		Dystonia	Myoclonus^a^	Ataxia	Other^b^	Unclassified	Total
Referral MD classification	Dystonia	26	1	0	4	1	32
	Myoclonus^a^	0	10	0	1	0	11
	Ataxia	0	8	0	1	0	9
	Other^b^	2	5	0	5	0	12
	Unclassified	13	7	1	12	3	36
	Total	41	31	1	23	4	100

^a^Isolated myoclonus, myoclonus ataxia and myoclonus dystonia

^b^Comprises chorea, tics, tremor, parkinsonism and if no MD was present

### Associated neurological and non-neurological features

Only 26/100 patients presented with a (mixed) MD without associated features, whereas the majority of patients also had additional neurological symptoms (*n* = 35), non-neurological symptoms (*n* = 9) or both (*n* = 30). The most important additional features were intellectual disability, epilepsy, spasticity, skin abnormalities, deafness, dysmorphias, and skeletal and growth abnormalities.

### Etiological diagnosis

At presentation, 29/100 patients had a confirmed genetic or acquired cause explaining their phenotype (Table 1). The multidisciplinary team established a diagnosis in 24 additional patients (Table 3), particularly in the genetic domain, where the number of diagnoses more than doubled from 17 to 37. Monogenetic etiologies were found using single-gene testing in nine cases, by targeted resequencing in three cases and using whole exome sequencing in five cases. Biochemical testing led to a diagnosis of non-ketotic hyperglycinemia in one case in which confirmation of the molecular defect is still pending.

**Table 3:** Confirmed etiological diagnoses after assessment by the multidisciplinary team

Diagnosis	***N***
Inherited etiologies	20
Monogenic	
*ACTB* mutation	1
*CTNNB1* mutation	1
*GLRA1* mutation	1
*GOSR2* mutation	6
*HSD17B10* mutation	1
*MECP2* mutation	1
*OFD-1* mutation	1
OTC-deficiency	1
*PRRT2* mutation	1
*SPTBN2* mutation	1
*TH* mutation	1
*TITF-1* mutation	1
Laboratory abnormalities	
Non-ketotic hyperglycinemia	1
Syndrome diagnosis	
Gilles de la Tourette	1
Linear naevus syndrome	1
Acquired etiologies	4
Drug-induced	1
Functional	3

*Abbreviations: ACTB*, beta-actin; *CTNNB1*, catenin (cadherin-associated protein) beta 1; *GLRA1*, glycine receptor alpha 1; *GOSR2*, Golgi SNAP receptor complex member 2; *HSD17B10*, 17beta-hydroxysteroid dehydrogenase type 10; *MECP2*, methyl CpG binding protein 2; *OFD-1*, oral-facial-digital syndrome 1; OTC, ornithine carbamoyltransferase; *PRRT2*, proline-rich transmembrane protein 2; *SPTBN2*, spectrin beta non-erythrocytic 2; TH, tyrosine hydroxylase; *TITF1*, thyroid transcription factor-1; HSD17B10 or 2-methyl-3-hydroxybytyryl-CoA dehydrogenase deficiency.

Among the acquired causes, oral contraceptive-induced chorea was diagnosed in one patient and three patients turned out to have functional MDs. Despite an increase in confirmed etiological diagnoses from 29 to 53, we still had 35 patients categorized with a suspected genetic diagnosis (defined as strong suspicion of a genetic cause based on a severe clinical phenotype, early onset, family history, and absence of any of the known acquired causes). In these cases, multiple genetic tests, including whole exome sequencing, have not yet revealed a causative molecular defect. For 21 of these 35 patients we are awaiting elucidation of the causal mutation in a research setting, the other 14 patients (or their caregivers) decided not to participate in this research.

### Treatment strategies

More than half of the 100 patients (61%) had not been given any specific treatment for their MD before visiting our clinic. The multidisciplinary team initiated or changed the treatment strategy in 60/100 of the patients. Table 4 gives an overview of changes in the treatment strategy, categorized by MD type. In 30/60 cases (50%), the new treatment strategy was based on the revised MD classification. In the other 30 patients the team initiated or adjusted the treatment strategy, despite an unchanged MD classification: for example symptomatic treatment with trihexyphenidyl in dystonic cerebral palsy. We advised six patients to stop their medication, which led to unchanged clinical symptoms in two patients and an improvement of symptoms in three others. An example of the latter was advice to stop taking oral contraceptives, which led to an almost complete disappearance of adolescent-onset chorea. In the group of 60 patients who had new or adjusted treatment, 72% of them or their caregivers reported a positive effect therapy after 3–6 months. Five patients were advised to stop their medication at the 3–6 months evaluation, because of limited benefit and or potential aggravation of other symptoms and side effects, such as effects on mood, behavior or constipation.

**Table 4:** Overview of treatment strategies that were changed by the multidisciplinary team

Movement disorder	Treatment category	Treatment specifics	*N*	Positive effect (*n)*
Dystonia				
	Pharmacological			
		Clonazepam	1	1
		Gabapentin	3	3
		L-dopa	2	1
		Trihexyphenidyl	8	3
		Cessation of drug	1	1
	Botulinum toxin		5	5
	Deep brain stimulation		5	4
	Paramedical		2	2
	Total dystonia		27	20
Myoclonus				
	Pharmacological	Clonazepam	10	10
	Ketogenic diet		4	1
	Paramedical		4	2
	Total myoclonus		18	12
Other				
	Pharmacological			
		L-dopa	4	4
		Acetozolamide	1	1
		Cessation of drug	4	2
	Botulinum toxin		1	1
	Paramedical		3	2
	Total other		13	10
Difficult to classify			2	
	Pharmacological	L-dopa	2	1
Total			60	43

## Discussion

To our knowledge, this is the first study describing the experience with a multidisciplinary team approach towards children and adults with YMDs. Based on the results it is likely that patients with YMDs benefit from a multidisciplinary team strategy with regard to MD classification, diagnostic yield and targeted treatment strategies.

The multifaceted nature of YMDs served as an impulse for setting up our multidisciplinary outpatient clinic, because the complexity of YMDs often leads to a time-consuming and burdensome diagnostic process [1, 6, 7]. This issue is reflected by a mean symptom duration of 74% of our patients’ life spans, which is in line with the results of a previous study [7]. In 58% of the patients, the team revised the MD classification or defined another MD as the most prominent clinical symptom. We think this high percentage of revisions may be due to the combined expertise of a pediatric neurologist, trained to distinguish normal developmental from abnormal movements, and a movement disorder specialist, trained to establish the phenomenology of clinical MD syndromes [1, 8]. Although we are aware that there is no gold standard for clinical MD classification, additional investigations such as EEG/EMG for myoclonus confirmed the clinical diagnosis in 24/29 of our cases [22]. The presence of non-neurological features in 39% of our YMD cohort underscores the complexity of the clinical presentations in a significant part of this population, and the combined expertise of a pediatrician and a clinical geneticist to include all symptoms, facilitated the diagnostic process.

The team identified a etiological diagnosis in 24/71 (34%) previously undiagnosed patients, of which 17 were found to have monogenetic disorders. In contrast, in a study with 260 patients non-tic YMDs patients, who were referred to a neurologist specialized in YMDs between 2004 and 2013, a definitive genetic diagnosis was made in 17%. [7]. We realize that the genetic advances of the past decade are likely to have contributed to the higher yield in our sample, however we hypothesize that the team’s broad and combined expertise has also been an important contributing factor. Furthermore, the diagnostic yield was obtained in a relatively short period of time, as a multidisciplinary team strategy facilitates immediate decision-making in comparison to the normal serial process involving multiple referrals, therefore minimizing the burden for the patients and their families.

After critical appraisal of phenotype and etiology, therapeutic strategies were considered and tailored to individual patient needs. The team gave specific advice on treatment for 60% of patients, with 72% (*n* = 42) of them or their caregivers reporting a subjective positive effect of the suggested treatment on follow-up. The effectiveness of treatment was only assessed through a semi-structured questionnaire and it was therefore not possible to draw more detailed conclusions on objective and/or long-term outcome measures of its effectiveness. Nevertheless, the large number of patients in which treatment was initiated at our clinic may reflect a potential under-treatment of YMDs, likely to significantly impact the patient’s quality of life. The low number of patients that were already treated for their MD is remarkable, in particular when taking into account that the mean MD severity of these 60 cases was significant (5 on a scale of 7). Low treatment rates and potential under-treatment have also been reported in MDs in children with inborn errors of metabolism, [13] despite the fact that it has been shown that symptomatic treatment may significantly improve patients’ daily functioning and quality of life [14, 23, 24].

The results of this exploratory study indicate that YMDs patients might benefit from a multidisciplinary team approach in terms of diagnosis and treatment in comparison to the referring specialists. However, interpretation of the results is limited by the lack of a control group of patients’ receiving assessment by a pediatric movement disorder specialist, or in comparison to assessments by an alternative team consisting of two or three specialists. Inclusion of such a control group was not feasible in our center. Nevertheless, we think that this single-institution experience indicates that a dedicated multidisciplinary approach to YMDs disorders may facilitate phenotyping and improve recognition of rare disorders.

Notably, in this study, the age at presentation at our outpatient clinic ranged from 1 to 33 years, which is beyond the standard upper limit of 18 years for pediatric care. Distinguishing early-onset from later-onset MDs is useful for diagnostic purposes [3, 4]. However, we believe that the age of symptom-onset in these patients is a more important inclusion criterion than the age at time of referral, especially because long delays between symptom-onset and diagnosis have been reported. [7] Therefore, we propose to consider patients with YMDs as a spectrum, irrespective of the age of referral, and to allow all complex YMD patients to benefit from the combined expertise of a multidisciplinary team, crossing barriers between pediatric and adult neurology.

## Conclusion

In summary, our results suggest that a multidisciplinary approach might help tackle the complexity of diagnosis and managing complex YMDs. Our experience indicates that this approach may improve recognition of rare disorders, with a good diagnostic yield and a minimal diagnostic delay. Future studies are needed to investigate the cost-benefit ratio of a multidisciplinary approach in comparison to regular subspecialty care, preferably using a prospective study design with standardized clinical assessments to systematically evaluate treatment effects.

## Abbreviations

EEG/EMG: Electroencephalography/electromyography
MD: Movement disorder
YMD: Young-onset movement disorder
